# 4-Meth­oxy-*N*′-(2-methoxy­naphthyl­idene)benzohydrazide

**DOI:** 10.1107/S1600536808026974

**Published:** 2008-08-23

**Authors:** Xiao-Yang Qiu

**Affiliations:** a Department of Chemistry, Shangqiu Normal University, Shangqiu 476000, People’s Republic of China

## Abstract

The mol­ecule of the title Schiff base compound, C_20_H_18_N_2_O_3_, prepared by the reaction of 2-meth­oxy-1-naphthyl­aldehyde and 4-methoxy­benzohydrazide, exists in a *trans* configuration with respect to the imine group. The naphthyl ring system makes a dihedral angle of 71.4 (2)° with the mean plane of the benzene ring. In the crystal structure, mol­ecules are linked into one-dimensional chains parallel to the *c* axis by inter­molecular N—H⋯O hydrogen bonds.

## Related literature

For the biological properties of hydrazone derivatives, see: Bedia *et al.* (2006 ▶); Rollas *et al.* (2002 ▶); Fun *et al.* (2008 ▶). For our previous reports of hydrazones, see: Qiu, Fang *et al.* (2006 ▶); Qiu, Luo *et al.* (2006*a* ▶,*b* ▶); Qiu, Xu *et al.* (2006 ▶). For related structures, see: Singh *et al.* (2007 ▶); Narayana *et al.* (2007 ▶); Cui *et al.* (2007 ▶); Diao *et al.* (2008 ▶).
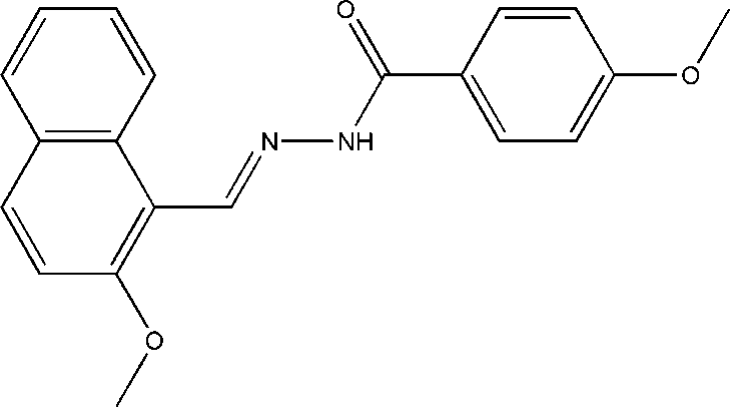


## Experimental

### 

#### Crystal data

C_20_H_18_N_2_O_3_
*M* *_r_* = 334.36
Monoclinic, 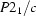

*a* = 11.675 (3) Å
*b* = 17.937 (4) Å
*c* = 8.508 (3) Å
β = 110.288 (3)°
*V* = 1671.2 (8) Å^3^
*Z* = 4
Mo *K*α radiation
μ = 0.09 mm^−1^
*T* = 298 (2) K
0.20 × 0.20 × 0.18 mm

#### Data collection

Bruker SMART CCD diffractometer
Absorption correction: multi-scan (*SADABS*;Sheldrick, 1996 ▶) *T*_min_ = 0.982, *T*_max_ = 0.984
9333 measured reflections
3445 independent reflections
1657 reflections with *I* > 2σ(*I*)
*R*_int_ = 0.048

#### Refinement

*R*[*F* ^2^ > 2σ(*F* ^2^)] = 0.055
*wR*(*F* ^2^) = 0.148
*S* = 0.97
3445 reflections
231 parameters
1 restraint
H atoms treated by a mixture of independent and constrained refinement
Δρ_max_ = 0.17 e Å^−3^
Δρ_min_ = −0.18 e Å^−3^

### 

Data collection: *SMART* (Bruker, 2001 ▶); cell refinement: *SAINT* (Bruker, 2001 ▶); data reduction: *SAINT*; program(s) used to solve structure: *SHELXS97* (Sheldrick, 2008 ▶); program(s) used to refine structure: *SHELXL97* (Sheldrick, 2008 ▶); molecular graphics: *SHELXTL* (Sheldrick, 2008 ▶); software used to prepare material for publication: *SHELXTL*.

## Supplementary Material

Crystal structure: contains datablocks I, global. DOI: 10.1107/S1600536808026974/bh2188sup1.cif

Structure factors: contains datablocks I. DOI: 10.1107/S1600536808026974/bh2188Isup2.hkl

Additional supplementary materials:  crystallographic information; 3D view; checkCIF report

## Figures and Tables

**Table 1:** Hydrogen-bond geometry (Å, °)

*D*—H⋯*A*	*D*—H	H⋯*A*	*D*⋯*A*	*D*—H⋯*A*
N2—H2⋯O2^i^	0.895 (10)	2.084 (12)	2.965 (3)	167 (2)

Symmetry code: (i) 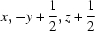.

**Fig. 1.**
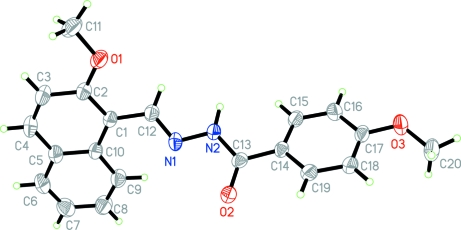
The molecular structure of (I), with atom labels and 30% probability displacement ellipsoids for non-H atoms.

**Fig. 2.**
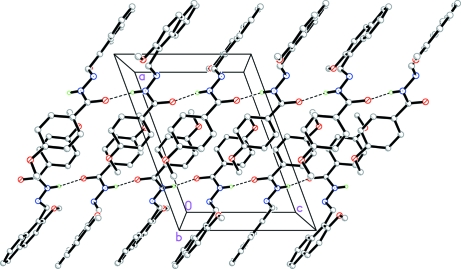
The crystal packing of (I), viewed along the b axis.

## supplementary crystallographic information

### Comment

Hydrazone compounds, which are derived from the reaction of aldehydes with hydrazides, have been widely studied due to their excellent biological properties (Bedia *et al.*, 2006; Rollas *et al.*, 2002; Fun *et al.*, 2008). Recently, we have reported a few Schiff hydrazone compounds (Qiu, Fang *et al.*, 2006; Qiu, Luo *et al.*, 2006*a*, 2006*b*; Qiu, Xu *et al.*, 2006), we report herein the crystal structure of the title new compound, (I).

The molecule of (I), Fig. 1, exists in a *trans* configuration with respect to the methylidene group. The naphthyl ring makes a dihedral angle of 71.4 (2)° with the mean plane of the benzene ring. The bond lengths and angles in (I) are found to have normal values and comparable to the values in similar compounds (Singh *et al.*, 2007; Narayana *et al.*, 2007; Cui *et al.*, 2007; Diao *et al.*, 2008).

In the crystal structure, molecules are linked into one-dimensional chains parallel to the *c* axis by intermolecular N—H···O hydrogen bonds (Table 1 and Fig. 2).

### Experimental

The title compound was prepared by the Schiff base condensation of equimolar (0.5 mmol each) 2-methoxy-1-naphthylaldehyde and 4-methoxybenzohydrazide in methanol (20 ml). Excess methanol was removed from the reaction mixture with distillation. The colourless solid was filtered and dried in air. Colourless block-shaped crystals suitable for X-ray diffraction were obtained from a methanol solution.

### Refinement

The imino H atom was located in a difference map and refined with N—H distance restrained to 0.90 (1) Å. The remaining H atoms were positioned geometrically [C—H = 0.93–0.96 Å] and refined using a riding model, with *U*_iso_(H) = 1.2*U*_eq_(C) and 1.5*U*_eq_(C_methyl_). Rigid rotating group models were used for the methyl groups.

### Crystal data

**Table:**

C_20_H_18_N_2_O_3_	*F*_000_ = 704
*M**_r_* = 334.36	*D*_x_ = 1.329 Mg m^−^^3^
Monoclinic, *P*2_1_/*c*	Mo *K*α radiation λ = 0.71073 Å
Hall symbol: -P 2ybc	Cell parameters from 1254 reflections
*a* = 11.675 (3) Å	θ = 2.5–24.5º
*b* = 17.937 (4) Å	µ = 0.09 mm^−^^1^
*c* = 8.508 (3) Å	*T* = 298 (2) K
β = 110.288 (3)º	Block, colourless
*V* = 1671.2 (8) Å^3^	0.20 × 0.20 × 0.18 mm
*Z* = 4	

### Data collection

**Table:**

Bruker SMART CCD diffractometer	3445 independent reflections
Radiation source: fine-focus sealed tube	1657 reflections with *I* > 2σ(*I*)
Monochromator: graphite	*R*_int_ = 0.048
*T* = 298(2) K	θ_max_ = 26.6º
ω scans	θ_min_ = 1.9º
Absorption correction: multi-scan(SADABS;Sheldrick, 1996)	*h* = −14→14
*T*_min_ = 0.982, *T*_max_ = 0.984	*k* = −22→18
9333 measured reflections	*l* = −10→10

### Refinement

**Table:**

Refinement on *F*^2^	Secondary atom site location: difference Fourier map
Least-squares matrix: full	Hydrogen site location: inferred from neighbouring sites
*R*[*F*^2^ > 2σ(*F*^2^)] = 0.055	H atoms treated by a mixture of independent and constrained refinement
*wR*(*F*^2^) = 0.148	*w* = 1/[σ^2^(*F*_o_^2^) + (0.0631*P*)^2^] where *P* = (*F*_o_^2^ + 2*F*_c_^2^)/3
*S* = 0.97	(Δ/σ)_max_ = 0.001
3445 reflections	Δρ_max_ = 0.17 e Å^−^^3^
231 parameters	Δρ_min_ = −0.18 e Å^−^^3^
1 restraint	Extinction correction: none
Primary atom site location: structure-invariant direct methods	

### Fractional atomic coordinates and isotropic or equivalent isotropic displacement parameters (Å^2^)

**Table:**

	*x*	*y*	*z*	*U*_iso_*/*U*_eq_	
O1	0.02930 (16)	0.01580 (9)	0.8049 (2)	0.0725 (6)	
O2	0.25127 (15)	0.32586 (9)	0.7183 (2)	0.0629 (5)	
O3	0.60192 (18)	0.45464 (11)	1.4125 (2)	0.0838 (6)	
N1	0.10006 (17)	0.22371 (11)	0.7604 (3)	0.0547 (6)	
N2	0.18996 (18)	0.25413 (11)	0.8945 (3)	0.0535 (6)	
C1	−0.0487 (2)	0.12593 (13)	0.6609 (3)	0.0492 (6)	
C2	−0.0564 (2)	0.04936 (14)	0.6724 (3)	0.0559 (7)	
C3	−0.1458 (3)	0.00788 (16)	0.5506 (4)	0.0722 (8)	
H3	−0.1477	−0.0438	0.5591	0.087*	
C4	−0.2290 (3)	0.04341 (17)	0.4209 (4)	0.0762 (9)	
H4	−0.2878	0.0154	0.3407	0.091*	
C5	−0.2297 (2)	0.12115 (16)	0.4033 (3)	0.0597 (7)	
C6	−0.3197 (3)	0.15768 (19)	0.2698 (4)	0.0765 (9)	
H6	−0.3786	0.1295	0.1899	0.092*	
C7	−0.3220 (3)	0.2322 (2)	0.2562 (4)	0.0818 (9)	
H7	−0.3827	0.2553	0.1684	0.098*	
C8	−0.2336 (3)	0.27484 (17)	0.3731 (4)	0.0790 (9)	
H8	−0.2356	0.3265	0.3630	0.095*	
C9	−0.1444 (2)	0.24203 (15)	0.5021 (4)	0.0662 (8)	
H9	−0.0859	0.2718	0.5782	0.079*	
C10	−0.1381 (2)	0.16348 (13)	0.5239 (3)	0.0504 (6)	
C11	0.0218 (3)	−0.06329 (14)	0.8242 (4)	0.0882 (10)	
H11A	−0.0584	−0.0761	0.8227	0.132*	
H11B	0.0813	−0.0785	0.9290	0.132*	
H11C	0.0374	−0.0883	0.7339	0.132*	
C12	0.0490 (2)	0.16441 (13)	0.7918 (3)	0.0542 (7)	
H12	0.0751	0.1457	0.9005	0.065*	
C13	0.2622 (2)	0.30763 (13)	0.8640 (4)	0.0504 (6)	
C14	0.3530 (2)	0.34273 (12)	1.0105 (3)	0.0486 (6)	
C15	0.3425 (2)	0.34574 (13)	1.1687 (3)	0.0562 (7)	
H15	0.2768	0.3225	1.1866	0.067*	
C16	0.4276 (3)	0.38248 (15)	1.2979 (3)	0.0658 (8)	
H16	0.4194	0.3830	1.4028	0.079*	
C17	0.5237 (2)	0.41823 (13)	1.2766 (4)	0.0612 (8)	
C18	0.5369 (2)	0.41555 (15)	1.1216 (4)	0.0670 (8)	
H18	0.6028	0.4390	1.1050	0.080*	
C19	0.4517 (2)	0.37779 (14)	0.9909 (3)	0.0593 (7)	
H19	0.4617	0.3761	0.8872	0.071*	
C20	0.6915 (3)	0.50102 (18)	1.3936 (4)	0.1010 (12)	
H20A	0.7404	0.4736	1.3433	0.151*	
H20B	0.7423	0.5192	1.5014	0.151*	
H20C	0.6536	0.5424	1.3230	0.151*	
H2	0.200 (2)	0.2348 (14)	0.9956 (19)	0.080*	

### Atomic displacement parameters (Å^2^)

**Table:**

	*U*^11^	*U*^22^	*U*^33^	*U*^12^	*U*^13^	*U*^23^
O1	0.0747 (13)	0.0456 (11)	0.0843 (15)	−0.0046 (9)	0.0111 (11)	0.0043 (10)
O2	0.0651 (12)	0.0541 (11)	0.0604 (13)	−0.0120 (8)	0.0104 (10)	0.0021 (9)
O3	0.0788 (14)	0.0771 (13)	0.0713 (14)	−0.0238 (11)	−0.0047 (11)	−0.0070 (11)
N1	0.0450 (12)	0.0532 (13)	0.0577 (14)	−0.0072 (10)	0.0072 (11)	−0.0104 (11)
N2	0.0515 (12)	0.0488 (13)	0.0524 (14)	−0.0103 (10)	0.0083 (11)	−0.0016 (11)
C1	0.0468 (15)	0.0476 (15)	0.0547 (17)	−0.0081 (12)	0.0193 (13)	−0.0066 (13)
C2	0.0513 (16)	0.0502 (16)	0.0646 (19)	−0.0060 (13)	0.0181 (15)	−0.0040 (14)
C3	0.0683 (19)	0.0537 (17)	0.089 (2)	−0.0161 (15)	0.0197 (18)	−0.0126 (16)
C4	0.068 (2)	0.069 (2)	0.081 (2)	−0.0206 (16)	0.0136 (18)	−0.0232 (17)
C5	0.0531 (17)	0.0646 (18)	0.0578 (18)	−0.0094 (14)	0.0146 (14)	−0.0110 (15)
C6	0.0603 (19)	0.094 (3)	0.063 (2)	−0.0087 (17)	0.0059 (15)	−0.0136 (18)
C7	0.070 (2)	0.092 (3)	0.069 (2)	0.0049 (18)	0.0060 (17)	0.0046 (19)
C8	0.0643 (19)	0.0676 (19)	0.090 (2)	0.0006 (16)	0.0075 (18)	0.0054 (17)
C9	0.0542 (17)	0.0577 (18)	0.075 (2)	−0.0040 (13)	0.0078 (15)	−0.0026 (15)
C10	0.0438 (14)	0.0504 (15)	0.0572 (17)	−0.0056 (12)	0.0177 (13)	−0.0069 (13)
C11	0.103 (3)	0.0471 (18)	0.109 (3)	−0.0049 (16)	0.030 (2)	0.0073 (17)
C12	0.0488 (15)	0.0477 (15)	0.0603 (17)	−0.0032 (12)	0.0115 (13)	−0.0016 (13)
C13	0.0508 (15)	0.0391 (14)	0.0590 (18)	0.0021 (12)	0.0160 (14)	0.0056 (13)
C14	0.0418 (14)	0.0382 (13)	0.0590 (18)	−0.0006 (11)	0.0090 (13)	0.0026 (12)
C15	0.0526 (15)	0.0515 (16)	0.0634 (19)	−0.0078 (12)	0.0188 (14)	0.0006 (14)
C16	0.0723 (19)	0.0597 (17)	0.0570 (18)	−0.0077 (15)	0.0117 (16)	−0.0060 (14)
C17	0.0516 (17)	0.0424 (15)	0.073 (2)	−0.0052 (12)	0.0000 (15)	0.0074 (14)
C18	0.0535 (17)	0.0699 (19)	0.068 (2)	−0.0162 (14)	0.0087 (15)	0.0117 (16)
C19	0.0534 (16)	0.0616 (16)	0.0616 (18)	−0.0061 (13)	0.0181 (14)	0.0078 (14)
C20	0.085 (2)	0.083 (2)	0.104 (3)	−0.0336 (19)	−0.006 (2)	−0.001 (2)

### Geometric parameters (Å, °)

**Table:**

O1—C2	1.361 (3)	C8—C9	1.358 (4)
O1—C11	1.434 (3)	C8—H8	0.9300
O2—C13	1.245 (3)	C9—C10	1.420 (3)
O3—C17	1.366 (3)	C9—H9	0.9300
O3—C20	1.388 (3)	C11—H11A	0.9600
N1—C12	1.292 (3)	C11—H11B	0.9600
N1—N2	1.368 (3)	C11—H11C	0.9600
N2—C13	1.361 (3)	C12—H12	0.9300
N2—H2	0.895 (10)	C13—C14	1.470 (3)
C1—C2	1.382 (3)	C14—C19	1.372 (3)
C1—C10	1.434 (3)	C14—C15	1.395 (3)
C1—C12	1.462 (3)	C15—C16	1.368 (3)
C2—C3	1.402 (4)	C15—H15	0.9300
C3—C4	1.351 (4)	C16—C17	1.358 (3)
C3—H3	0.9300	C16—H16	0.9300
C4—C5	1.402 (4)	C17—C18	1.381 (4)
C4—H4	0.9300	C18—C19	1.384 (3)
C5—C6	1.413 (4)	C18—H18	0.9300
C5—C10	1.419 (3)	C19—H19	0.9300
C6—C7	1.341 (4)	C20—H20A	0.9600
C6—H6	0.9300	C20—H20B	0.9600
C7—C8	1.388 (4)	C20—H20C	0.9600
C7—H7	0.9300		
			
C2—O1—C11	118.4 (2)	O1—C11—H11A	109.5
C17—O3—C20	119.9 (3)	O1—C11—H11B	109.5
C12—N1—N2	115.6 (2)	H11A—C11—H11B	109.5
C13—N2—N1	118.0 (2)	O1—C11—H11C	109.5
C13—N2—H2	124.7 (18)	H11A—C11—H11C	109.5
N1—N2—H2	117.2 (18)	H11B—C11—H11C	109.5
C2—C1—C10	118.5 (2)	N1—C12—C1	121.7 (2)
C2—C1—C12	117.9 (2)	N1—C12—H12	119.2
C10—C1—C12	123.6 (2)	C1—C12—H12	119.2
O1—C2—C1	116.8 (2)	O2—C13—N2	121.3 (2)
O1—C2—C3	121.5 (2)	O2—C13—C14	121.8 (2)
C1—C2—C3	121.7 (3)	N2—C13—C14	116.9 (2)
C4—C3—C2	119.6 (3)	C19—C14—C15	117.3 (2)
C4—C3—H3	120.2	C19—C14—C13	118.9 (2)
C2—C3—H3	120.2	C15—C14—C13	123.8 (2)
C3—C4—C5	122.2 (3)	C16—C15—C14	120.7 (2)
C3—C4—H4	118.9	C16—C15—H15	119.6
C5—C4—H4	118.9	C14—C15—H15	119.6
C4—C5—C6	121.5 (3)	C17—C16—C15	121.6 (3)
C4—C5—C10	118.7 (3)	C17—C16—H16	119.2
C6—C5—C10	119.8 (3)	C15—C16—H16	119.2
C7—C6—C5	121.3 (3)	C16—C17—O3	117.0 (3)
C7—C6—H6	119.4	C16—C17—C18	118.8 (3)
C5—C6—H6	119.4	O3—C17—C18	124.1 (3)
C6—C7—C8	120.0 (3)	C17—C18—C19	119.8 (3)
C6—C7—H7	120.0	C17—C18—H18	120.1
C8—C7—H7	120.0	C19—C18—H18	120.1
C9—C8—C7	120.8 (3)	C14—C19—C18	121.7 (3)
C9—C8—H8	119.6	C14—C19—H19	119.1
C7—C8—H8	119.6	C18—C19—H19	119.1
C8—C9—C10	121.8 (3)	O3—C20—H20A	109.5
C8—C9—H9	119.1	O3—C20—H20B	109.5
C10—C9—H9	119.1	H20A—C20—H20B	109.5
C5—C10—C9	116.4 (2)	O3—C20—H20C	109.5
C5—C10—C1	119.3 (2)	H20A—C20—H20C	109.5
C9—C10—C1	124.2 (2)	H20B—C20—H20C	109.5

### Hydrogen-bond geometry (Å, °)

**Table:**

*D*—H···*A*	*D*—H	H···*A*	*D*···*A*	*D*—H···*A*
N2—H2···O2^i^	0.895 (10)	2.084 (12)	2.965 (3)	167 (2)

Symmetry codes: (i) *x*, −*y*+1/2, *z*+1/2.

## References

[bb1] Bedia, K.-K., Elçin, O., Seda, U., Fatma, K., Nathaly, S., Sevim, R. & Dimoglo, A. (2006). *Eur. J. Med. Chem.* **41**, 1253–1261. 10.1016/j.ejmech.2006.06.009 16919372

[bb2] Bruker (2001). *SMART* and *SAINT* Bruker AXS Inc., Madison, Wisconsin, USA.

[bb3] Cui, J., Yin, H. & Qiao, Y. (2007). *Acta Cryst.* E**63**, o3548.

[bb4] Diao, Y.-P., Zhen, Y.-H., Han, X. & Deng, S. (2008). *Acta Cryst.* E**64**, o101. 10.1107/S1600536807062861 PMC2915176 21200667

[bb5] Fun, H.-K., Patil, P. S., Jebas, S. R., Sujith, K. V. & Kalluraya, B. (2008). *Acta Cryst.* E**64**, o1594–o1595. 10.1107/S1600536808022861 PMC2962208 21203289

[bb6] Narayana, B., Siddaraju, B. P., Raju, C. R., Yathirajan, H. S. & Bolte, M. (2007). *Acta Cryst.* E**63**, o3522.

[bb7] Qiu, X.-Y., Fang, X.-N., Liu, W.-S. & Zhu, H.-L. (2006). *Acta Cryst.* E**62**, o2685–o2686.

[bb8] Qiu, X.-Y., Luo, Z.-G., Yang, S.-L. & Liu, W.-S. (2006*a*). *Acta Cryst.* E**62**, o3531–o3532.

[bb9] Qiu, X.-Y., Luo, Q.-Y., Yang, S.-L. & Liu, W.-S. (2006*b*). *Acta Cryst.* E**62**, o4291–o4292.

[bb10] Qiu, X.-Y., Xu, H.-J., Liu, W.-S. & Zhu, H.-L. (2006). *Acta Cryst.* E**62**, o2304–o2305.

[bb11] Rollas, S., Gülerman, N. & Erdeniz, H. (2002). *Farmaco*, **57**, 171-174. 10.1016/s0014-827x(01)01192-2 11902660

[bb12] Sheldrick, G. M. (1996). *SADABS* University of Göttingen, Germany.

[bb13] Sheldrick, G. M. (2008). *Acta Cryst.* A**64**, 112–122. 10.1107/S0108767307043930 18156677

[bb14] Singh, N. K., Singh, M., Srivastava, A. K., Shrivastav, A. & Sharma, R. K. (2007). *Acta Cryst.* E**63**, o4895. 10.1107/S1600536807067037 PMC2915380 21200900

